# Contributors to the black-white life expectancy gap in Washington D.C.

**DOI:** 10.1038/s41598-020-70046-6

**Published:** 2020-08-27

**Authors:** Max Roberts, Eric N. Reither, Sojung Lim

**Affiliations:** Department of Sociology, Social Work and Anthropology, Utah State University, Logan, UT 84322 USA

**Keywords:** Epidemiology, Diseases, Risk factors

## Abstract

Although the black-white gap in life expectancy has been shrinking in the U.S., national improvement conceals ongoing disparities. Nowhere is this more evident than Washington D.C., where the black-white gap has persistently exceeded 10 years. Using 1999–2017 mortality data from the National Center for Health Statistics, we employed demographic techniques to pursue three aims: first, we created period life tables to examine longevity trends in Washington D.C.; second, we decomposed black-white life expectancy differences into 23 causes of death in three time periods (2000, 2008, 2016); third, we assessed age-specific contributions for each cause of death. Findings revealed that heart disease (4.14 years), homicide (2.43 years), and cancer (2.30 years) contributed most to the 17.23-year gap among males in 2016. Heart disease and cancer contributed most at ages 55–69; homicide contributed most at ages 20–29. Among females in 2016, heart disease (3.24 years), cancer (2.36 years), and unintentional injuries (0.85 years) contributed most to the 12.06-year gap. Heart disease and cancer contributed most at ages 55–69, and unintentional injuries at ages 50–59. Our investigation provides detailed evidence about contributors to the black-white longevity gap in Washington D.C., which can aid in the development of targeted public health interventions.

## Introduction

The black-white gap in life expectancy in the United States is currently about 3.6 years, which is a 50% reduction from 1970^1,2^. This promising national trend conceals disparities across U.S. states and municipalities that are less encouraging^3,4^. For example, Harper et al.^4^ found that black-white life expectancy gaps increased among males in Alaska, Hawaii, Maine, New Hampshire, Wisconsin, and the District of Columbia (a.k.a., Washington D.C.) between 1990 and 2009. They also found increasing black-white gaps among females in Nebraska, Wisconsin, and Washington D.C. One particularly notable finding from this study was the large black-white life expectancy gap in Washington D.C.—14.70 years among males and 10.60 years among females in 2009^4^. In fact, the black-white life expectancy gap in Washington D.C. was larger than any other state or territory in the nation, and nearly twice the size of the second largest gap in Wisconsin. In addition to all 50 states, the black-white longevity gap in D.C. exceeds those of peer cities. For example, in 2015 black-white longevity gaps among males were 1.03 years in New York City, 3.57 years in Philadelphia, and 9.52 years in San Francisco^5^. As we will show, the black-white life expectancy gap in Washington D.C. has unfortunately continued to grow in recent years, warranting further investigation.

Washington D.C. has a total population of just over 700,000 and is composed of 46.4% black residents. The percentage of black residents is greater in Washington D.C. than any U.S. state, including Mississippi (37.8%) and Louisiana (32.7%)^6^. For comparative purposes, the black population of about 325,000 in Washington D.C. is larger than the *entire* populations of major U.S. cities like St. Paul in Minnesota, Pittsburgh in Pennsylvania, and Cincinnati in Ohio. Given the large size of the black population and the small geographic area that places all Washington D.C. residents in relatively close proximity, it is imperative to understand why black residents face vastly different longevity prospects than white residents.

Although the black-white longevity gap in Washington D.C. is an urgent public health concern, there is limited evidence that policymakers can draw upon to combat this stark disparity. One recent study assessed how five broadly-defined causes of death (cardiovascular disease, cancer, non-communicable disease, communicable disease, and injury) contributed to the black-white life expectancy gap across all U.S. states from 1969–2013^7^. This study found that cardiovascular disease and cancer made the greatest contributions to the black-white life expectancy gap in Washington D.C. in 2013, with injuries also making a large contribution among males. This study successfully identified key contributors, but large portions of the gap were not attributed to particular causes. Moreover, it is important to disentangle some broad categories (e.g., injuries) used in this study into more specific causes (e.g., homicide, vehicle accidents, and drug overdoses) to help stakeholders identify potential solutions.

By following the approach used in a recent study of Wisconsin^8^, we will show how various causes of death contribute to the black-white longevity gap in Washington D.C. Because non-Hispanics are distinct from Hispanics in terms of socioeconomic factors and related health outcomes^9^, we focus on mortality disparities between non-Hispanic blacks (hereafter, blacks) and non-Hispanic whites (hereafter, whites). The aims of our investigation are threefold: first, we calculate life expectancies for males and females in Washington D.C. and the U.S. from 2000 to 2016, revealing how black-white gaps in Washington D.C. have diverged from national trends; second, we decompose longevity gaps into 23 specific causes of death at three different periods of time (2000, 2008, and 2016); third, we assess each cause of death across 19 different age categories, showing how each cause of death contributes to black-white disparities over the life course. The overarching motivation for our investigation is to provide new, detailed evidence about contributors to the black-white longevity gap in Washington D.C., which can be used to create targeted interventions and public health policies.

## Results

### Aim 1: Tracing black-white disparities since 2000 in Washington D.C. and the U.S

**Figure 1:**
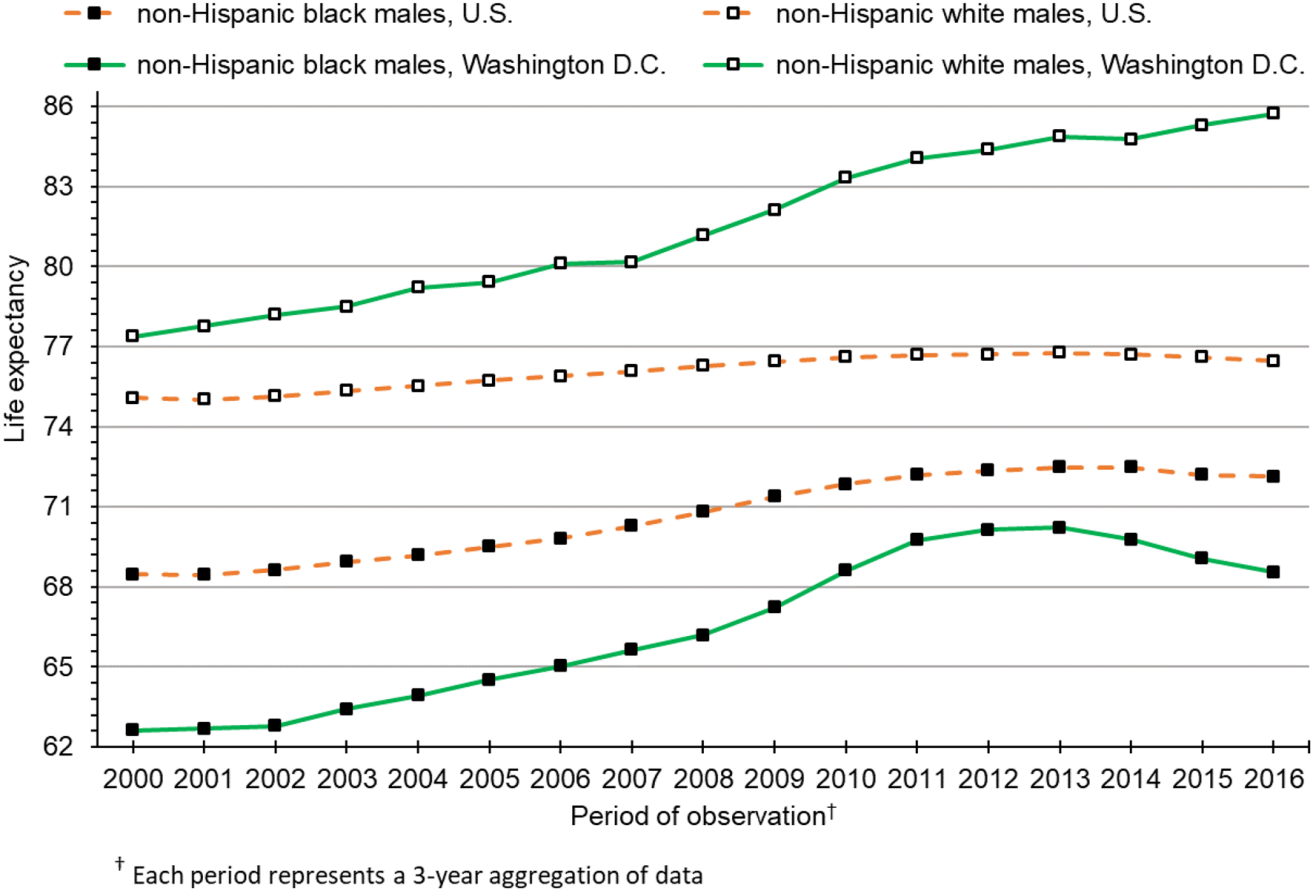
Life expectancy trends in the U.S. and Washington D.C. for non-Hispanic black and non-Hispanic white males.

Figure 1 presents life expectancy trends for black and white males in the U.S. and Washington D.C. from 2000 to 2016. In 2000, the national black-white gap for males was 6.60 years. At 14.68 years, the corresponding gap in Washington D.C. was more than twice as large. Over the past two decades, the national black-white longevity gap has steadily declined among males, reaching 4.32 years in 2016. Conversely, the gap in Washington D.C. was fairly stable in the early 2000s but diverged sharply after 2012, reaching 17.23 years in 2016. The black-white gap of 17.23 years among males in Washington D.C. was 399% larger than the black-white gap among U.S. males.

**Figure 2:**
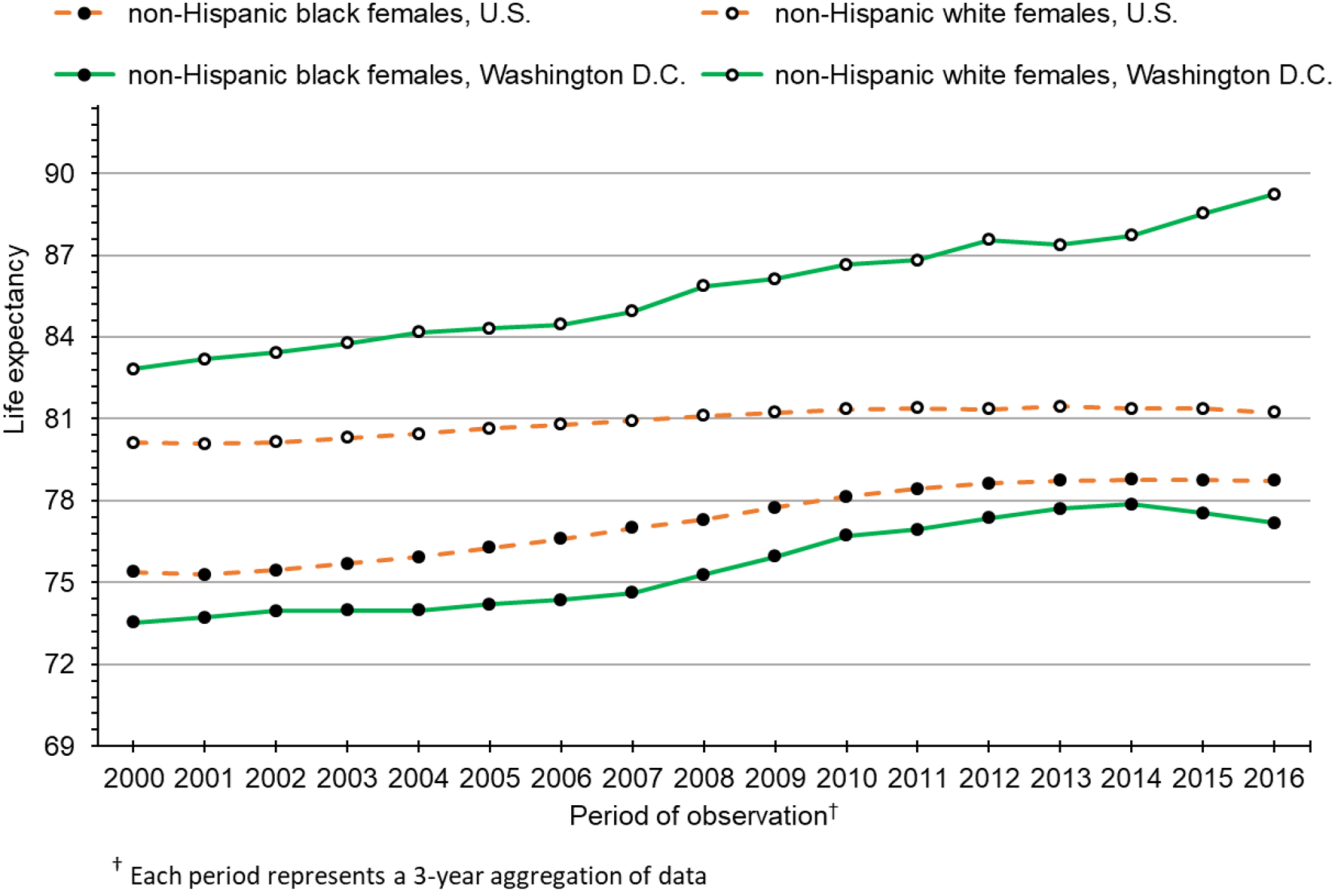
Life expectancy trends in the U.S. and Washington D.C. for non-Hispanic black and non-Hispanic white females.

Figure 2 presents life expectancy trends for black and white females in the U.S. and Washington D.C. As we observed among males, the black-white gap for U.S. females has converged over time, reaching an all-time low of 2.50 years in 2016. During our study period, both black and white females in Washington D.C. experienced substantial gains in life expectancy. However, gains among white females outpaced black females and, by 2016, the gap between them had grown to 12.06 years. This gap of 12.06 years was 482% larger than the corresponding black-white gap among U.S. females.

Shortly after the Great Recession of 2008, life expectancy stagnated for U.S. males and females from both race groups. Conversely, white males and females in Washington D.C. continued to experience substantial gains in life expectancy after 2009. Similarly, life expectancy continued to rise after the Great Recession among black males and females in Washington D.C. However, progress among blacks halted around 2013 for males and 2014 for females. Since that time, black life expectancy in Washington D.C. has declined. Continuing gains in life expectancy among white males in Washington D.C., coupled with recent declines in life expectancy among black males, have led to rapid growth in the black-white gap. From 2013 to 2016, the gap grew from 14.64 to 17.23 years, an increase of 2.59 years over a span of just 3 years. Similar trends among females in Washington D.C. have also resulted in widening of the black-white longevity gap—from 9.86 years in 2014 to 12.06 years in 2016, an increase of 2.20 years.

### Aim 2: Contribution of 23 causes of death to black-white longevity gaps

Table 1 presents the contribution of 22 selected causes of death and a residual “all other causes” of death category to the black-white life expectancy gap for males at three different time periods. We found that heart disease (4.14 years), homicide (2.43 years), malignant neoplasms (2.30 years), and unintentional injuries (2.23 years) contributed most to the 17.23-year gap in 2016. We disaggregated malignant neoplasms into eight types of cancer, revealing that lung cancer (0.52 years) made the largest contribution to the gap, followed by colorectal cancer (0.31 years), liver cancer (0.30 years), and prostate cancer (0.29 years). Disaggregating the unintentional injuries category indicated a large contribution from drug poisoning (1.56 years), followed by motor vehicle accidents (0.36 years). Diabetes, perinatal conditions, and HIV were other notable causes of death among males, each contributing over half a year to the black-white gap in 2016. Perhaps our most notable finding was the large contribution of homicide to the black-white gap, which exceeded the total contribution of cancer in each study period. Another interesting finding was the change in key contributors over time among males. Between 2000 and 2016, the contribution of heart disease, unintentional injuries, and cancer to the black-white gap increased by a combined total of 3.81 years. In addition, the contribution of diabetes more than doubled from 2000 (0.29 years) to 2016 (0.69 years), becoming the fifth largest contributor to the black-white gap. Conversely, the combined contribution of HIV and homicide decreased by 1.80 years over our study period.

**Table 1:** Contribution of 23 causes of death (in years) to life expectancy (*e*_0_) differences between non-Hispanic black and non-Hispanic white males in Washington D.C.

Cause of death	2000	2008	2016	Change between 2000 and 2016	Percent of *e*_0_ gap, 2016
All cancer (malignant neoplasms)	1.70	1.94	2.30	0.60	13.3
Colorectal	0.09	0.20	0.31	0.22	1.8
Esophageal	0.18	0.11	0.03	− 0.15	0.2
Liver	0.06	0.24	0.30	0.24	1.7
Lung	0.63	0.52	0.52	− 0.11	3.0
Pancreatic	0.02	0.16	0.13	0.11	0.7
Prostate	0.23	0.31	0.29	0.05	1.7
Stomach	0.08	0.12	0.07	− 0.01	0.4
All other cancers	0.41	0.29	0.66	0.25	3.8
Cerebrovascular disease	0.27	0.35	0.42	0.15	2.5
Diabetes	0.29	0.38	0.69	0.40	4.0
Heart disease	2.14	2.84	4.14	2.00	24.0
HIV	1.67	1.38	0.59	− 1.08	3.4
Homicide	3.15	3.00	2.43	− 0.72	14.1
Hypertension	0.13	0.15	0.28	0.15	1.6
Influenza and pneumonia	0.14	0.11	0.21	0.07	1.2
Liver disease	0.23	0.16	0.19	− 0.04	1.1
Nephritis	0.19	0.16	0.23	0.04	1.3
Perinatal conditions	0.65	0.43	0.51	− 0.14	3.0
Respiratory disease	0.18	0.13	0.34	0.16	2.0
All unintentional injuries	1.02	0.84	2.23	1.21	13.0
Drug poisoning	0.35	0.21	1.56	1.21	9.0
Motor vehicle accidents	0.35	0.36	0.36	0.01	2.1
All other unintentional injuries	0.31	0.27	0.32	0.01	1.9
All other causes	2.95	3.12	2.67	− 0.28	15.5
Total e_0_ difference	14.70	15.01	17.23		

Table 2 presents the contribution of selected causes of death to the black-white life expectancy gap for females in 2000, 2008, and 2016. Our analyses indicate that heart disease (3.24 years), cancer (2.36 years), unintentional injuries (0.85 years), perinatal conditions (0.57), and diabetes (0.55 years) contributed most to the 12.06-year gap in 2016. We found that breast cancer (0.43 years) was the largest cancer contributor, followed by lung cancer (0.38 years) and colorectal cancer (0.28 years). In the unintentional injuries category, drug poisoning (0.65 years) again contributed more to the gap than motor vehicle accidents (0.12 years). Cerebrovascular disease and HIV were also important contributors among females, accounting for 0.40 and 0.35 years of the gap in 2016, respectively. Altogether, heart disease and cancer accounted for 81.9% of the 2.77-year increase in the black-white gap among females between 2000 and 2016. Whereas the contribution of drug poisoning increased by almost half a year over this timeframe, the contribution of HIV declined by just over half a year.

**Table 2:** Contribution of 23 causes of death (in years) to life expectancy (*e*_0_) differences between non-Hispanic black and non-Hispanic white females in Washington D.C.

Cause of death	2000	2008	2016	Change between 2000 and 2016	Percent of e_0_ gap, 2016
All cancer (malignant neoplasms)	1.22	1.95	2.36	1.14	19.6
Breast	0.16	0.30	0.43	0.27	3.6
Colorectal	0.25	0.25	0.28	0.03	2.3
Esophageal	0.06	0.04	0.01	− 0.05	0.1
Liver	0.05	0.09	0.13	0.08	1.1
Lung	0.21	0.45	0.38	0.17	3.2
Pancreatic	0.09	0.15	0.19	0.10	1.6
Stomach	0.07	0.07	0.05	− 0.02	0.4
All other cancers	0.34	0.60	0.89	0.55	7.4
Cerebrovascular disease	0.35	0.36	0.40	0.05	3.3
Diabetes	0.75	0.53	0.55	− 0.20	4.6
Heart disease	2.11	2.41	3.24	1.13	26.9
HIV	0.88	1.01	0.35	− 0.53	2.9
Homicide	0.30	0.30	0.26	− 0.04	2.1
Hypertension	0.11	0.12	0.20	0.09	1.7
Influenza and pneumonia	0.07	0.06	0.14	0.07	1.1
Liver disease	0.09	0.19	0.13	0.04	1.1
Nephritis	0.20	0.17	0.24	0.04	2.0
Perinatal conditions	0.82	0.62	0.57	− 0.25	4.7
Respiratory disease	− 0.04	0.06	0.32	0.36	2.7
All unintentional Injuries	0.27	0.36	0.85	0.58	7.1
Drug poisoning	0.19	0.10	0.65	0.46	5.4
Motor vehicle accidents	− 0.01	0.13	0.12	0.13	1.0
All other unintentional injuries	0.09	0.13	0.08	− 0.01	0.7
All other causes	2.16	2.41	2.44	0.28	20.2
Total e_0_ difference	9.29	10.55	12.06		

To characterize within-race changes in life expectancy from 2000 to 2016, we conducted a supplemental series of decomposition analyses. During this period, life expectancy increased by 5.91 years among black males and 3.64 years among black females (Supplementary Table S1). Reductions in HIV, all cancer, and homicide mortality accounted for 61.3% of the 5.91-year gain among black males. This gain was partly offset by drug poisoning deaths, which reduced life expectancy gains by 0.72 years. Among black females, improvements in heart disease contributed most (1.29 years) to the 3.64-year gain in life expectancy, followed by HIV (0.61 years), all cancer (0.48 years), and diabetes (0.46 years). Drug poisoning prevented the gain among black females from being larger, accounting for a 0.45-year loss.

Between 2000 and 2016, life expectancy increased by 8.44 years among white males and 6.41 years among white females (Supplementary Table S2). Improvements in heart disease (2.74 years) and all cancer (2.36 years) made the largest contributions to life expectancy gains among white males, followed by HIV (0.50 years) and respiratory disease (0.32 years). White females experienced notable improvements in heart disease (2.83 years), all cancer (1.76 years), and respiratory disease (0.39 years). Although increases in drug poisoning were less dramatic among whites, life expectancy gains in all four race-sex groups were suppressed by drug poisoning between 2000 and 2016.

### Aim 3: Life stages that contribute most to the black-white gap in life expectancy

**Figure 3:**
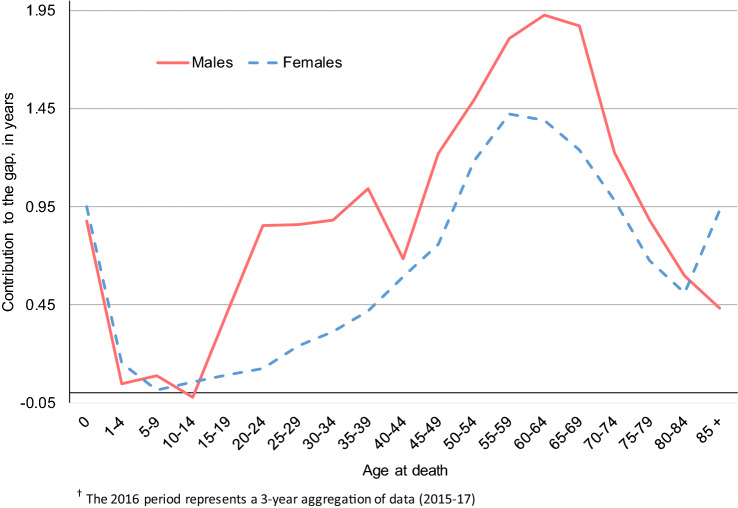
Age-specific contributions to black-white gaps in life expectancy among non-Hispanic males and females, 2016^†^

Figure 3 illustrates how each age group contributed to the black-white life expectancy gap for males and females in 2016. For both males and females, the earliest life stage (< 1 year of age) contributed nearly a year to the black-white gap in life expectancy. Ages 1 to 14 contributed relatively little to the black-white gap for both sexes. Beginning with the age group 15–19, black-white disparities rose sharply among males, increasing to approximately one year at age 35–39. After a temporary decrease at age 40–44, the male black-white gap grew again, peaking at nearly two years at age 60–64. For females, the black-white gap in life expectancy increased steadily after age 15, reaching a maximum of almost 1.5 years at age 55–59. Contributions to the gap quickly tapered off among males and females after age 65. However, in the oldest age group (85+), the black-white gap among females surged again to nearly a year. To summarize, among males the largest contributions to the black-white gap occurred before age 1, from age 15 to 39, and from age 45 to 74. Among females, the largest contributions occurred before age 1 and from age 40 to 74.

**Figure 4:**
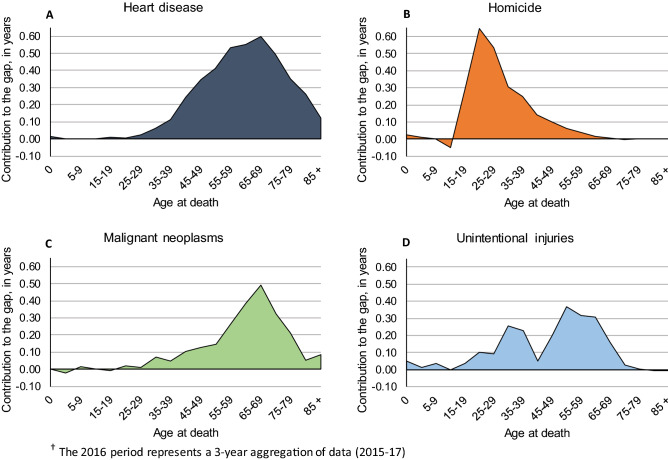
Age and cause decomposition of Washington D.C.’s black-white gap in life expectancy among non-Hispanic males, 2016^†^

Figure 4 shows age-specific distributions for the four leading contributors to the black-white longevity gap among males in 2016. We elected to focus on these causes of death, as age-specific contributions to the black-white gap were relatively small for other causes. As shown in panels A and C of Fig. 4, heart disease and cancer made large contributions to the black-white gap at older life stages. Heart disease made the greatest contribution to the gap at ages 55–59 (0.53 years), 60–64 (0.55 years), and 65–69 (0.60 years). Similarly, cancer made the greatest contribution at ages 60–64 (0.38 years), 65–69 (0.49 years), and 70–74 (0.32 years). The contribution of homicide to the black-white longevity gap (Fig. 4, panel B) was heavily concentrated among adolescent and young adult males. The spike at age 20–24 indicates that homicide made a 0.65-year contribution to the gap in this age group alone. Homicide also made large contributions to the gap among males at ages 15–19 (0.29 years), 25–29 (0.53 years), and 30–34 (0.31 years). Panel D of Fig. 4 indicates that unintentional injuries made moderate contributions to the gap between age 30 and 65.

**Figure 5:**
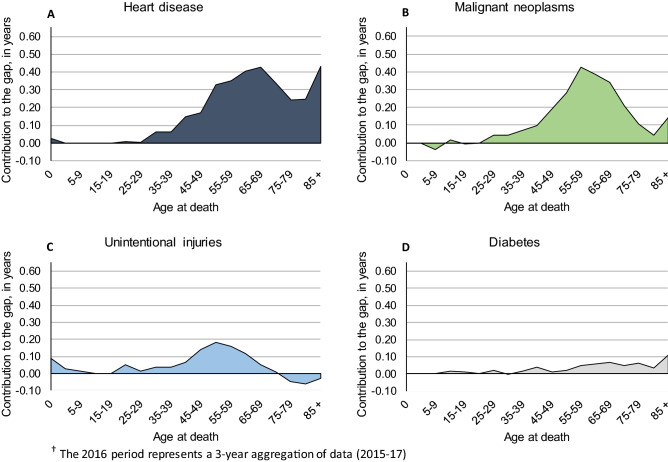
Age and cause decomposition of Washington D.C.’s black-white gap in life expectancy among non-Hispanic females, 2016^†^

For females, we illustrate age- and cause-specific contributions to the black-white life expectancy gap in Fig. 5. Note that we omit perinatal conditions (the fourth leading contributor) and include diabetes instead (the fifth leading contributor), as perinatal conditions occur entirely in the first year of life. As we found among males, heart disease (Fig. 5, panel A) and cancer (Fig. 5, panel B) contributed most to the female black-white gap at later stages of life. Heart disease contributed most after age 55; the two largest contributions occurred at ages 65–69 and 85+ (0.43 years in each age group). Cancer’s peak contribution occurred at age 55–59 (0.43 years) among females, with a fairly steep drop-off before and after that age. As shown in panel C of Fig. 5, unintentional injuries made notable contributions at ages 45–49 (0.14 years), 50–54 (0.18 years), and 60–64 (0.12 years). Diabetes (Fig. 5, panel D) contributed most at ages 65–69 (0.07 years) and 85+ (0.11 years).

## Discussion

Our investigation revealed disconcerting trends in the black-white life expectancy gap in Washington D.C. In the most recent period of observation (2016), black males could expect to live 17.23 years less than white males, and black females could expect to live 12.06 years less than white females. The longevity gap in Washington D.C. has widened considerably in recent years, as white life expectancy has continued to increase and black life expectancy has begun to decrease. If recent trends continue, black and white life expectancies will diverge to an even greater extent in the future.

Heart disease was the leading contributor to the black-white longevity gap in 2016. Moreover, racial disparities in heart disease mortality widened between 2000 and 2016 among both males and females. The rising contribution of heart disease could be related to obesity, physical activity, and smoking disparities in Washington D.C. Although overweight and obesity increased among all race-sex groups, the rising prevalence in obesity (i.e., BMI ≥ 30) and morbid obesity (i.e., BMI ≥ 40) among black D.C. residents is of special concern, given health risks associated with this condition. Data from the Behavioral Risk Factor Surveillance System (BRFSS)^10^ show that the prevalence of obesity was > 2 times higher among black males than white males in both 2000 and 2016. Additionally, morbid obesity rose sharply among black males, from 2.3% in 2000 to 4.2% in 2016. By comparison, morbid obesity was low among white males—0.6% in 2000 and 0.7% in 2016. Among black females, the prevalence of obesity increased markedly, from 29.6% in 2000 to 37.4% in 2016. Over this period, obesity among white females increased only slightly, from 6.2% in 2000 to 7.2% in 2016. The prevalence of morbid obesity was > 10 times higher among black females in both time periods. It is well established that obesity increases the risk of developing various diseases of the heart. Public health interventions such as education about healthful diet, food labeling, advertisement restrictions, and incentivizing SNAP recipients to purchase healthy foods could help reduce black-white obesity disparities in Washington D.C^11^.

In 2016, 26% of black D.C. residents reported that they did not engage in physical activity or exercise in the past month, compared to 6.1% of white residents^12^. Disadvantaged individuals may face barriers to exercise such as insufficient time and feelings of exhaustion^13^. In addition, many people rely on safe sidewalks and public parks for exercise, but these amenities are often lacking in predominantly black neighborhoods^14^. Additional funds and strategic zoning in disadvantaged neighborhoods could create new resources, including walking and biking trails. Additionally, requiring physical education in schools may improve regular physical activity among blacks in Washington D.C. at early ages^13^.

Smoking is a major risk factor for ischemic heart disease, which leads to atherosclerosis and increased risk of heart failure and heart attack^15^. Although the prevalence of smoking among black males in Washington D.C. decreased from 25.8% in 2000 to 21.6% in 2016, it was much higher than white males (15.6% in 2000 and 10.0% in 2016)^12^. Black females also experienced a modest decline in smoking prevalence, from 21.7% in 2000 to 17.4% in 2016. Although smoking prevalence among white females was only slightly lower than black females in 2000 (17.7%), it declined sharply to 7% in 2016^12^. Tobacco advertising heavily targets neighborhoods with large black populations^16^. Reducing smoking prevalence in black neighborhoods could be achieved through tobacco price increases, mass-media anti-smoking campaigns, smoke-free policies, restricting tobacco marketing, and educating youths about smoking risks in school^17^. Because this is not a prospective cohort study, we cannot definitively link disparities in smoking, physical activity, and obesity to the mortality disparities that we observed. However, extant research on these risk factors is sufficiently strong to warrant public health interventions designed to reduce racial disparities in these health behaviors^15,18^.

In addition to heart disease, cancer was an increasingly large contributor to black-white longevity gaps in Washington D.C. between 2000 and 2016. Increasing contributions of breast cancer among females, prostate cancer among males, and colorectal cancer among both sexes point to shortcomings in screening and early detection. Because black residents of Washington D.C. often lack access to early detection screening, they are generally diagnosed with cancer at later stages of the disease^19^. Insufficient knowledge about preventive healthcare among black and other minority populations is another driver for underutilization of breast, colorectal, and prostate cancer screening^20^. Educational initiatives to disseminate health information, such as the use of lay health advisors^21^, could improve knowledge of breast, colorectal, and prostate cancer risks and early-detection screening among blacks in Washington D.C.

Lung cancer, another major contributor to the black-white longevity gap, is strongly associated with tobacco use. As noted, smoking prevalence is substantially higher among blacks in Washington D.C. Public health campaigns to reduce smoking prevalence among blacks could reduce the incidence of lung cancer, thereby shrinking its contribution to the black-white longevity gap. As discussed, these interventions could include restrictions on tobacco advertising, increased tax on tobacco products, smoking risk education in schools, and smoke-free policies.

By examining a wider array of causes than previous research, we discovered that homicide was the second largest contributor to the black-white longevity gap among males, accounting for more of the gap than cancer. Adolescent and young adult black males between age 15 and 34 were particularly vulnerable to homicide. In 2017, black males in Washington D.C. faced a homicide rate of 61.5 per 100,000, which was the fourth highest homicide rate among black males in the nation, behind only Missouri, Illinois, and Indiana^22^. The high risk of homicide for young black males is an important contributor to reduced life expectancy, as premature deaths during adolescence and young adulthood reduce the potential person-years lived in the black male population.

A silver lining in our findings is that homicide’s contribution to the black-white longevity gap has declined substantially among males in Washington D.C. over the past two decades. Another promising finding among males was the sharp decline in HIV, which contributed a year less to the black-white gap in 2016 than in 2000. Among females, the contribution of homicide remained relatively small and stable across our period of study, and the contribution of HIV decreased by over half a year. Despite the high rate of homicide in Washington D.C., it has trended downward in recent years^23^. We can see these improvements clearly in supplementary table S1, as reductions in homicide among males and HIV among both sexes has led to life expectancy gains between 2000 and 2016.

Reduced homicide in Washington D.C. is attributable in part to community outreach and police initiatives like the Gun Recovery Unit and the Summer Crime Initiative, which focus all resources in the summer months to districts that experience high rates of violent crime^23^. Other effective homicide interventions include individual behavior change through improving educational success or enhancing knowledge and skills to avoid expressive violence^24^. Engaging at-risk individuals, including youths arrested during violent incidents or gunshot wound survivors, and providing them with appropriate resources could further reduce the risk of homicide mortality^25^.

Reductions in HIV are also attributable in part to successful public health policies. In particular, the 90–90–90–50 plan was implemented in 2005, which aimed to end the HIV epidemic in Washington D.C. by 2020. Since its implementation, Washington D.C. has seen a 72% drop in new HIV infections, from a high of 1,343 incident cases in 2007 to just 371 in 2015^26^. This is a promising decline, particularly for blacks who make up nearly 70% of all people living with HIV in Washington D.C^26^.

Unintentional injuries were the third largest contributor to the black-white longevity gap among females, and the fourth leading contributor among males. Since 2000, the contribution of unintentional injuries has increased substantially, especially among males. This increase is largely attributable to the opioid epidemic and accidental drug poisoning. In 2017, Washington D.C. had 244 overdose deaths involving opioids, which translates into a rate of 34.7 deaths per 100,000 persons^27^. This rate ranked as third highest in the U.S. and more than twice the national average of 14.6 deaths per 100,000^27^. Blacks in Washington D.C. have been especially burdened by the opioid epidemic, as they experienced 216 deaths in 2017—a rate of 60 deaths per 100,000—which was a higher rate of opioid overdose mortality than any other group of whites, blacks, or Hispanics in the entire U.S^28^. Opioid overdose mortality among blacks in Washington D.C. is a public health priority; addressing this issue would result in immediate reductions to the black-white longevity gap.

Diabetes and perinatal conditions were other notable contributors, each accounting for more than half a year of black-white gaps in life expectancy among males and females. BRFSS data show that diabetes prevalence among black males in Washington D.C. increased from 8.2% in 2000 to 12.5% in 2016. Similarly, diabetes prevalence among black females increased from 12.3% in 2000 to 16.6% in 2016. Although diabetes prevalence also increased among white males and females in Washington D.C., reflecting the general rise in overweight and obesity^10^, racial disparities in diabetes are nevertheless stark. As of 2016, fewer than 3% of white males or females in Washington D.C. reported diabetes. Reducing diabetes prevalence among black D.C. residents and improving access to diabetes treatments will reduce racial disparities in longevity.

High rates of black infant mortality are another concern in Washington D.C. In 2016, the black infant mortality rate in Washington D.C. was 11.4 per 1,000 live births, which was nearly nine deaths greater than whites in Washington D.C. (2.5 per 1,000)^29,30^. Disparities in infant mortality rates likely reflect underlying socioeconomic inequalities, as well as differential access to quality healthcare^31^. For example, during 2015–2016, 86% of white D.C. mothers received prenatal care in their first trimester, but nearly 40% of black D.C. mothers did not receive prenatal care until their second or third trimester; over 4% of blacks received no prenatal care at all^32^. Differences in smoking prevalence may also play a role in black-white disparities in infant mortality. Recent data from Washington D.C. indicate that nearly 5% of black mothers smoked during pregnancy, compared to fewer than 1% of white mothers^32^.

Black mothers in Washington D.C. are also at high risk of maternal mortality. Although we omitted maternal mortality from our final analyses because it contributed a relatively small amount to the black-white longevity gap (0.05 years in 2016), we note that maternal mortality among black females in Washington D.C. is the worst in the nation (59.7 death per 100,000 live births)^33^. Meanwhile, white females in Washington D.C. have the lowest maternal mortality ratio in the U.S., suggesting that while excellent maternal care is available in Washington D.C., it is not accessible to all residents^34^.

In 2018, Washington D.C. boasted the second lowest percentage of uninsured residents at 3.2%, behind only Massachusetts^35^. Although the percentage of uninsured non-elderly blacks (4%) and whites (3%) was similar^36^, 54% of black residents in Washington D.C. received public health insurance, which was the second highest public insurance rate for blacks in the nation^37^. Conversely, only 13% of whites in Washington D.C. were covered by public insurance^37^. Different sources of healthcare coverage reflect unequal socioeconomic conditions for blacks and whites in Washington D.C., and they are likely indicative of differential access to the highest standards of care.

Black-white disparities in health care and health outcomes in Washington D.C. may be largely attributable to fundamental social and economic causes^38^. Washington D.C. is highly segregated, with blacks representing just 5–10% of the population in western parts of the city and more than 90% in communities east of the Anacostia River^31^. Racial segregation is associated with environmental hazards (e.g., air and noise pollution, lead paint, and asbestos), high crime rates, poor quality schools, and food deserts. Consistent with those markers of segregation, the Washington D.C. Department of Health has highlighted nine key drivers of public health equity, including education, employment, income, housing, transportation, food environment, medical care, outdoor environment, and community safety^39^. While careful study of these factors is beyond the scope of this investigation, it is important to recognize that they may underlie black-white longevity disparities. Equitable access to effective education, rewarding employment opportunities, safe neighborhoods and housing, and high-quality health care would likely reduce black-white health disparities in Washington D.C.

Another factor that may have increased black-white longevity disparities in Washington D.C. is the in-migration of select whites. From 2000 to 2017, the white population increased from 30 to 41%^40^. Over this timeframe, the magnitude of the socioeconomic gap between whites and blacks is evident in median household incomes. In 2007, black and white D.C. residents had median household incomes of about $42,000 and $116,000, respectively^41^. By 2017, median income among white households in Washington D.C. increased to over $160,000, compared to just $48,000 among black households^42^. Whites in Washington D.C. also have more education than their black counterparts. In 2000, over 80% of whites had a bachelor’s degree, compared to 17% of blacks^43^. By 2017, over 88% of whites had a bachelor’s degree or higher, compared to 26.7% of blacks^44^.

A notable limitation of our investigation is that our mortality data are not paired with socioeconomic indicators. Consequently, although we discuss social and economic disadvantages faced by blacks in Washington D.C., we are unable to quantify how much these factors contribute to the black-white longevity gap. Our study also does not examine the impact that gentrification and mobility have on the black-white longevity gap. Since 2000, the white population in Washington D.C. has become more select (as noted), but the black population has declined by 16%.^45^ Between 1990 and 2010, black-majority tracts decreased from 67 to 57%, and white-majority tracts increased from 26 to 30%. Gentrification is a serious social problem in Washington D.C. as it leads to widespread displacement of black residents^45^, potentially exacerbating longstanding socioeconomic and health disparities. Future research would benefit from evaluating the effect of gentrification and migration on widening black-white longevity disparities.

Despite these limitations, a notable strength of our study is the use of restricted-access data, which provide complete uncensored mortality counts, thus eliminating the need for sophisticated imputation estimates, as have been used previously with public-use data^4,7^. Additionally, our investigation used the latest data available and expanded the focus to 23 different causes of death, leading to several new discoveries. Another strength is our assessment of multiple age groups, highlighting stages in the life course where each cause of death contributed most to black-white gaps in life expectancy.

The growing black-white life expectancy gap in Washington D.C. is an urgent public health priority. Our findings affirmed that heart disease and cancer are major contributors to the gap in mid-to-late life. Among males, we found that homicide contributes more to the gap than cancer, afflicting adolescents and young adults most. Unintentional injuries also made a considerable contribution to the gap among males in mid-to-late life, and among females in later life. Growing contributions of heart disease, cancer, and unintentional injuries (particularly drug overdoses) have driven recent increases in the black-white gap. Reducing homicide among young black males and drug poisoning among black males and females—and easing the burden of chronic conditions such as heart disease and cancer among older blacks—will help Washington D.C. achieve its stated public health goal of health equity.

## Methods

### Data

Our investigation utilized restricted-use multiple cause of death-all county micro data files for 1999–2017^46^, granted to us by National Center for Health Statistics (NCHS). We used data on death counts (*D*_*ij*_) and population estimates (*N*_*ij*_) to calculate death rates (*M*_*ij*_ = *D*_*ij*_/*N*_*ij*_) for selected subgroups (*i*) and periods of observation (*j*) in Washington D.C. Restricted-use NCHS data provide complete, uncensored mortality counts for total and specific causes of death by period of observation, sex, age, ethnicity, and race. Following prior work^4^, we used U.S. Census Bureau population estimates with bridged race categories as denominators^47^. The population estimates cover the same time periods and demographic characteristics as the complete mortality counts.

### Measures

As noted, we restricted our analyses to non-Hispanics, as they differ from Hispanics with respect to socioeconomic characteristics and health outcomes^9^. We also stratified our analyses by sex as cause-specific death rates vary between males and females^48^. Our study focused on four race-sex groups: non-Hispanic black males, non-Hispanic black females, non-Hispanic white males, and non-Hispanic white females. For each of these race-sex groups, we conducted a series of life table analyses. We categorized age in each life table as follows: (1) less than one year of age, (2) 1–4 years of age, (3) a series of five-year age groups ranging from 5–9 to 80–84, and (4) an open-ended category for ages 85 and older.

We selected 23 causes of death previously identified as leading causes of death nationwide^49^, or as suspected contributors to the black-white life expectancy gap^50^. These causes of death include breast cancer (among females), colorectal cancer, esophageal cancer, liver cancer, lung cancer, pancreatic cancer, prostate cancer (among males), stomach cancer, all other cancers, cerebrovascular disease, diabetes, heart disease, HIV, assault (homicide), hypertension, influenza and pneumonia, liver disease, nephritis, perinatal conditions, respiratory disease, drug poisoning, motor vehicle accidents, all other unintentional injuries, and a residual category for all remaining causes of death. We disaggregated malignant neoplasms into different types of cancer, as they are heterogeneous in their etiology, prevention, and treatment. Additionally, we disaggregated unintentional injuries into drug poisoning and motor vehicle accidents, which are the two leading causes of unintentional death^51^. Causes of death were categorized in accordance with the International Classification of Diseases (ICD), 10th revision^52^, and coded using the Department of Vital Statistics Underlying Cause of Death 358 Recode^53^ (see Supplementary Table S3 for cause of death coding scheme).

### Analysis

We used Microsoft Excel 2013 for our analyses, which aggregated three years of death (*D*_*ij*_) and population (*N*_*ij*_) data into individual cross-sections of time to minimize random fluctuations in mortality rates (*M*_*ij*_) for each age-race-sex subgroup (e.g., 2000 represents 1999–2001). To address study aim 1 (tracing black-white disparities since 2000), we converted age-race-sex mortality rates into probability estimates and generated period life tables for each group (i.e., black and white males and females) in Washington D.C. as well as the entire U.S. In the process of converting rates to probabilities, we employed graduation techniques to estimate the average person-years lived among individuals who died between the ages of *x* to *x* + *n* (_*n*_*a*_*x*_)^54^. Next, we ordered life expectancies (*e*_0_) derived from these life tables into three-year moving averages, spanning the time period 2000 (i.e., 1999–2001) to 2016 (i.e., 2015–2017). These analyses facilitated visualization of trends in Washington D.C.’s black-white life expectancy gap, relative to the rest of the nation.

To address aim 2 (identifying causes of death that contributed most to black-white disparities), we decomposed the overall black-white *e*_0_ gap into portions attributable to 22 sex-specific causes of death, as well as a residual category for all other causes of death. We employed the age and cause decomposition method with discrete data following prior work^7,55,56^. Addressing our final aim (identifying life stages where specific causes of death made the greatest contributions), we calculated the number of years that each cause of death contributed to the overall black-white *e*_0_ gap for each age group. We assessed aims 2 and 3 at three different time points spanning our entire period of observation: 2000 (1999–2001), 2008 (2007–2009), and 2016 (2015–2017).

## Supplementary information

Supplementary Information

## Data Availability

Data supporting the findings of this study are available from the National Center for Health Statistics. However, restrictions apply to the availability of these data, which were used under license for the current study, and so are not publicly available. Data are however available from the National Center for Health Statistics with a restricted-use data agreement.
