# Supplementary material for: Contributors to the black-white life expectancy gap in Washington D.C
Source: Sci Rep. 2020 Aug 27;10:13416. doi: 10.1038/s41598-020-70046-6 (PMC7453009; doi:10.1038/s41598-020-70046-6)
Supplement: Supplementary file 1 — Supplementary Information [file 41598_2020_70046_MOESM1_ESM.docx]

**Contributors to the Black-White Life Expectancy Gap in Washington D.C.**

Max Roberts${}^{1}$, Eric N. Reither${}^{1}$, Sojung Lim${}^{1}$

${}^{1}$ Utah State University, Department of Sociology, Social Work & Anthropology, Logan, UT, 84322, U.S.

Supplementary Table S1. Contribution of 23 causes of death (in years) to life expectancy (*e_0_*) change among non-Hispanic black males and females from 2000 to 2016 in Washington D.C.

| Cause of death | Males | Females |
| --- | --- | --- |
| All cancer (malignant neoplasms) | 1.20 | 0.48 |
| Breast | -- | 0.11 |
| Colorectal | 0.04 | 0.11 |
| Esophageal | 0.16 | 0.02 |
| Liver | -0.10 | -0.05 |
| Lung | 0.49 | 0.23 |
| Pancreatic | -0.02 | -0.05 |
| Prostate | 0.18 | -- |
| Stomach | 0.06 | 0.06 |
| All other cancers | 0.38 | 0.06 |
| Cerebrovascular disease | 0.10 | 0.16 |
| Diabetes | -0.03 | 0.46 |
| Heart disease | 0.51 | 1.29 |
| HIV | 1.34 | 0.61 |
| Homicide | 1.08 | 0.12 |
| Hypertension | -0.05 | -0.04 |
| Influenza and pneumonia | 0.04 | 0.04 |
| Liver disease | 0.13 | 0.01 |
| Nephritis | 0.07 | 0.07 |
| Perinatal conditions | 0.34 | 0.32 |
| Respiratory disease | 0.06 | -0.10 |
| All unintentional injuries | -0.52 | -0.55 |
| Drug poisoning | -0.72 | -0.45 |
| Motor vehicle accidents | 0.17 | -0.02 |
| All other unintentional injuries | 0.03 | -0.09 |
| All other causes | 1.64 | 0.77 |
| Total e_0_ change | **5.91** | **3.64** |

Note: Table entries represent the contribution, in years, to the total change in life expectancy. Some of these values may be negative, reflecting suppression of life expectancy gains between 2000 and 2016.

Supplementary Table S2. Contribution of 23 causes of death (in years) to life expectancy (*e_0_*) change among non-Hispanic white males and females from 2000 to 2016 in Washington D.C.

| Cause of death | Males | Females |
| --- | --- | --- |
| All cancer (malignant neoplasms) | 2.36 | 1.76 |
| Breast | -- | 0.44 |
| Colorectal | 0.36 | 0.16 |
| Esophageal | 0.06 | -0.05 |
| Liver | 0.06 | 0.02 |
| Lung | 0.59 | 0.53 |
| Pancreatic | 0.09 | 0.00 |
| Prostate | 0.25 | -- |
| Stomach | 0.06 | 0.01 |
| All other cancers | 0.90 | 0.65 |
| Cerebrovascular disease | 0.29 | 0.29 |
| Diabetes | 0.26 | 0.12 |
| Heart disease | 2.74 | 2.83 |
| HIV | 0.50 | 0.09 |
| Homicide | 0.16 | 0.05 |
| Hypertension | -0.03 | -0.03 |
| Influenza and pneumonia | 0.11 | 0.18 |
| Liver disease | 0.11 | 0.06 |
| Nephritis | 0.07 | 0.03 |
| Perinatal conditions | 0.20 | 0.06 |
| Respiratory disease | 0.32 | 0.39 |
| All unintentional injuries | -0.04 | -0.15 |
| Drug poisoning | -0.13 | -0.12 |
| Motor vehicle accidents | 0.15 | 0.12 |
| All other unintentional injuries | -0.06 | -0.15 |
| All other causes | 1.39 | 0.72 |
| Total e_0_ change | **8.44** | **6.41** |

Note: Table entries represent the contribution, in years, to the total change in life expectancy. Some of these values may be negative, reflecting suppression of life expectancy gains between 2000 and 2016.

Supplementary Table S3. Department of Vital Statistics Underlying Cause of Death 358 Recode coding scheme

| Cause of death | 358 Recode coding scheme |
| --- | --- |
| All cancer (malignant neoplasms) |  |
| Breast | 104 |
| Colorectal | 081-083 |
| Esophageal | 77 |
| Liver | 085-086 |
| Lung | 93 |
| Pancreatic | 88 |
| Prostate | 113 |
| Stomach | 78 |
| All other cancers | 072-075; 079; 087; 089; 091-092; 094-096; 098-099; 101-103; 106-111; 114-115; 117-120; 122-124; 125; 127; 129-132; 134-137; 139-140; 142-146 |
| Cerebrovascular disease | 235-239 |
| Diabetes | 159 |
| Heart disease | 199; 201-204; 207; 209; 211-215; 218-219; 221-222; 224-228; 230-233 |
| HIV | 049-053 |
| Homicide | 433-441 |
| Hypertension | 206; 208 |
| Influenza and pneumonia | 253; 255-257 |
| Liver disease | 298-302 |
| Nephritis | 323-325; 327; 329 |
| Perinatal conditions | 357-364 |
| Respiratory disease | 264-269 |
| All unintentional injuries |  |
| Drug poisoning | 420 |
| Motor vehicle accidents | 386-388; 390-398 |
| All other unintentional injuries | 384; 399-402; 404-418; 421-423 |
| All other causes | All else |
